# StralSV: assessment of sequence variability within similar 3D structures and application to polio RNA-dependent RNA polymerase

**DOI:** 10.1186/1471-2105-12-226

**Published:** 2011-06-02

**Authors:** Adam T Zemla, Dorothy M Lang, Tanya Kostova, Raul Andino, Carol L Ecale Zhou

**Affiliations:** 1 Global Security Computing Applications Division, Lawrence Livermore National Laboratory, Livermore, CA 94550, USA; 2 National Science Foundation, Arlington, VA 22230, USA; 3 Department of Microbiology and Immunology, University of California, San Francisco, CA 94143, USA

## Abstract

**Background:**

Most of the currently used methods for protein function prediction rely on sequence-based comparisons between a query protein and those for which a functional annotation is provided. A serious limitation of sequence similarity-based approaches for identifying residue conservation among proteins is the low confidence in assigning residue-residue correspondences among proteins when the level of sequence identity between the compared proteins is poor. Multiple sequence alignment methods are more satisfactory--still, they cannot provide reliable results at low levels of sequence identity. Our goal in the current work was to develop an algorithm that could help overcome these difficulties by facilitating the identification of structurally (and possibly functionally) relevant residue-residue correspondences between compared protein structures.

**Results:**

Here we present StralSV (structure-alignment sequence variability), a new algorithm for detecting closely related structure fragments and quantifying residue frequency from tight local structure alignments. We apply StralSV in a study of the RNA-dependent RNA polymerase of poliovirus, and we demonstrate that the algorithm can be used to determine regions of the protein that are relatively unique, or that share structural similarity with proteins that would be considered distantly related. By quantifying residue frequencies among many residue-residue pairs extracted from local structural alignments, one can infer potential structural or functional importance of specific residues that are determined to be highly conserved or that deviate from a consensus. We further demonstrate that considerable detailed structural and phylogenetic information can be derived from StralSV analyses.

**Conclusions:**

StralSV is a new structure-based algorithm for identifying and aligning structure fragments that have similarity to a reference protein. StralSV analysis can be used to quantify residue-residue correspondences and identify residues that may be of particular structural or functional importance, as well as unusual or unexpected residues at a given sequence position. StralSV is provided as a web service at http://proteinmodel.org/AS2TS/STRALSV/.

## Background

Accurate sequence alignments between related proteins are important for many bioinformatics applications that involve comparative analysis. Derived from calculated alignments, residue-residue correspondences allow construction of sequence motifs and profiles important in building homology models or in predicting protein functions. Most of the currently used methods for protein function prediction rely on sequence-based comparisons between a query protein and those for which a functional annotation is provided. A serious limitation of sequence similarity-based approaches for identifying residue conservation among proteins is the lack of, or very low, confidence in assigning residue-residue correspondences among proteins when the level of sequence identity between the compared proteins is poor. Indeed, it was shown by Rost [1] that more than 95% of all pair-wise alignments occurring in the so-called twilight zone (20-35% sequence identity) may be incorrect [2]. Multiple sequence alignment methods are more satisfactory--still, they cannot provide reliable results at low levels of sequence identity, especially if the number of available closely related proteins is small (i.e., when the protein family has rather few members, or the list of related proteins that has been identified is short).

Having 3D structural information for a given protein can be especially useful in deriving functional annotation [3]. Structure comparison algorithms provide much higher confidence in assignment of residue-residue correspondences than do sequence-based algorithms. Nevertheless even calculated structural alignments may be inaccurate: for some compared proteins, or regions therein, more than one possible superposition can reasonably be reported, and it may be difficult to decide which alignment is most satisfactory [4,5]. Rigid body structural superpositions on the chain level have limitations when comparing multi-domain proteins with different conformations between domains. Comparisons on the domain level may yield better results, but splitting of structures into domains can be problematic, and there is no reliable method that can do this automatically. Even within compared structural domains, significant deviations can be observed in some loop regions, or due to large insertions or different conformations of structural motifs, all of which can significantly affect detection of structural residue-residue correspondences when rigid body approaches are used for alignment calculations. Several algorithms have been proposed to facilitate flexible protein structure alignment calculations [6-8], but the complexity of such calculations remains a challenging development goal. Another difficulty in identifying similar regions in compared protein structures lies in the possibility that analogous regions in structurally related proteins may display differences in sequential ordering of the motifs due to circular permutations or convergent evolution [9,10]. The majority of the existing flexible protein structure alignment algorithms report only sequential alignments, and there are very few (with varying levels of success) that can detect and align structures between which there are differences in the ordering of their structure motifs [11-13].

The accuracy of calculated structural alignments can also depend on the nature of compared structural models. The atomic coordinates obtained from experimentally solved structures (x-ray crystallography or nuclear magnetic resonance spectroscopy) are always associated with some degree of uncertainty resulting from experimental errors from the intrinsic flexibility of the proteins or from atom vibrations [14]. Such structural deviations may sometimes significantly affect the calculated alignments and lead to incorrect conclusions about sequence motifs, profiles, or possible residue substitutions within analyzed functional regions in proteins. The accuracy of calculated residue-residue correspondences can be improved by refinement methods that evaluate results produced by different structure-based alignment programs or explore sequence-based alignments using, for example, the Conserved Domain Database (CDD) as a set of reference alignments [15].

Our goal in the current work was to develop an algorithm that could address these difficulties and facilitate the identification of structurally (and possibly functionally) relevant residue-residue correspondences between compared protein structures. Our approach is to first detect similar structural motifs, and consequently derive structure-based alignments from the calculated local superpositions of corresponding similar regions. Our StralSV algorithm detects structurally similar regions within a given pair of protein structures, and reports residue-residue correspondences only from those local regions that are contained within a larger, similar structural context. When for a given reference structure a structure-based search is performed on a set of proteins from the Protein Data Bank (PDB), StralSV identifies all structurally similar fragments from that set, evaluates the calculated structure-based alignments between the query (reference) motif (designated "segment" in this work) and the detected structure fragments, and quantifies the observed sequence variability at each residue position on the query structure. Here we describe how the StralSV algorithm works, and we apply StralSV in a study of the RNA-dependent RNA polymerase of poliovirus.

## Methods

### Description of the StralSV algorithm

**Figure 1:**
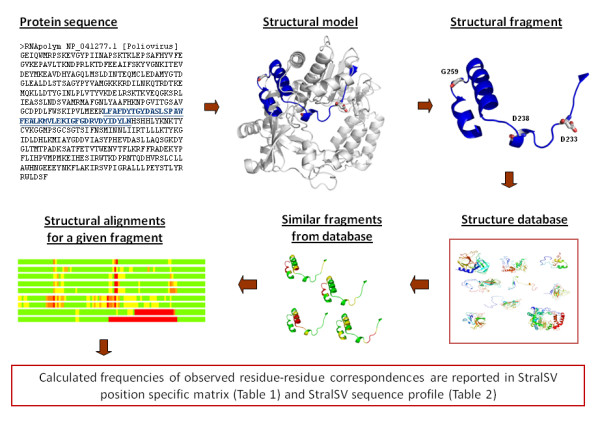
**Overview of the process and data types used in the StralSV algorithm**.

StralSV is an algorithm that identifies protein structural fragments having a 3D structure similar to that of a query structure, performs structure-based alignments between the query and the fragments, and quantifies at each position along the query structure the sequence variability represented among the selected fragments relative to the query. StralSV takes as input a query structure of interest, a database of protein structures, and various parameters (discussed below) that control the selection of fragments from the database and the sequence variability calculations. Figure 1 illustrates the steps in the algorithm. The algorithm uses a sliding-window approach for breaking the query structure into overlapping segments, each of which is independently used to identify from the input database protein structure fragments with 3D similarity. A recommended (default) window_size parameter is set to 90 amino acids in length, although an arbitrary length can be chosen. The query structure is thus split into overlapping segments of length window_size; overlaps are by default 1/2 the length of the window_size. A final segment is taken one window_size in length extending from the C-terminus to ensure that all portions of the query structure are represented within a segment of exactly the window_size. Each so-calculated query segment is then compared to all protein structures in the database using the LGA (local-global alignment) code [16] to identify structure fragments with sufficient structure similarity to the query segment. The LGA_S score is used to evaluate structure similarity between a query segment and detected similar fragments. Calculated LGA_S scores range from 0% to 100% and reflect a percentage of residues from the query segment that are identified as structurally aligned with a given similar fragment. In the StralSV algorithm, a value of LGA_S of at least 50% is used as a cutoff to ensure that there is sufficient structural similarity between the segment and the fragment over at least half the length of the segment. LGA's distance cutoff parameter determines the maximum allowable distance between alpha carbons (Cα) of superimposed amino acids within a calculated alignment; typically this parameter is set to 4.0 Å, and this default is used for StralSV calculations with window_size values of 90 residues. Thus, fragments with sufficient structure similarity to the query segment are identified. Each fragment is then evaluated to determine the tightness of its alignment to the query segment.

The criteria for tight structure similarities in local regions (spans), described by Zemla et al. [17], are used to identify ranges within the alignment that have tight local superpositions. Each residue-residue pair from the alignment (closest superimposed residues from the query segment and database fragment) is assigned a score by calculating the local RMSD (root mean square deviation) among the surrounding residue-residue pairs. A continuous set of at least three residue-residue pairs that fulfil the RMSD cutoff of 0.5 Å comprises a span. A desired size of a calculated span (span_size; shortest acceptable tight local alignment without gaps) is used as an input parameter to StralSV, and is typically specified as 3, 5, or 7, but can be of arbitrary length. Our previous experience [17], suggests that 5 is a reasonable minimum length over which to impose a tight alignment; 3 is the minimum value for span_size that is meaningful (since any 2 *Cα *atoms can be perfectly aligned), and 7 imposes stringency that tends to eliminate capture of some related (desired) structure fragments. For the work reported here we selected a minimum span length of 5 (span_size = 5). From each alignment is extracted a set of spans. All alignments that contain at least one span of length no less than the specified minimum span length are deemed "qualified hits". (For an illustration of a "span", see additional file 1: StralSV-RdRp_Suppl_Figure 1.docx.) All residue-residue pairs that are contained within a span's alignment are used to calculate the sequence variability data at the corresponding position in the query structure. Note that not all residues from calculated structure alignments contribute to the variability statistics at a given position in the query segment; regions in which the local RMSD distances between corresponding residues exceed 0.5 Å induce breakpoints between spans. Also, because the algorithm uses overlapping segments, duplications are appropriately factored out in calculating the sequence variability.

A frequency matrix (Table 1) is constructed for each position in the query structure by tallying the frequency at which each amino-acid (the 20 standard amino acids plus 'X', corresponding to unusual or modified residues) is observed within the spans. From this matrix are extracted statistics describing the number of positional hits (residue-residue correspondences contributing sequence variability data) per position and a list of residues observed at each position, ordered by frequency of occurrence. These statistics are used to construct a variability profile (Table 2), which can be used to identify positions at which there is relatively high or low sequence variability in structure context. The sample profile given in Table 2 shows the observed residues for positions 229 through 269 for polio RNA-dependent RNA polymerase (RdRp) run as the query protein against the complete PDB database (released on November 24, 2009).

**Table 1:** Excerpt of sample StralSV output "matrix" file for positions 229-269 from poliovirus RdRp analysis

AA	Rname	A	V	L	I	P	M	F	W	G	S	T	C	Y	N	Q	D	E	K	R	H	X
L	229	4	0	21	10	0	0	1	0	103	0	0	0	0	0	0	0	0	0	0	4	0
F	230	0	4	4	3	0	8	127	0	0	0	0	0	5	0	0	0	0	0	0	0	4
A	231	30	0	0	0	0	0	0	0	0	116	0	2	5	0	0	4	0	0	0	0	0
F	232	8	10	7	4	0	0	34	0	0	0	49	0	140	0	0	5	0	0	0	0	0
D	233	0	0	0	0	0	0	0	0	0	0	0	0	0	0	0	272	0	0	0	0	0
Y	234	0	49	4	11	0	0	0	0	0	0	152	0	60	0	0	0	0	0	0	0	0
T	235	5	0	0	0	0	0	0	0	0	91	25	0	0	0	0	0	4	7	144	0	0
G	236	23	0	0	0	0	0	0	0	23	0	0	139	0	13	5	48	0	11	13	0	1
Y	237	4	0	0	0	0	0	152	37	4	0	0	7	28	0	0	0	0	0	0	44	0
D	238	0	0	0	0	0	0	0	0	0	0	0	0	4	0	0	268	4	0	0	0	0
A	239	41	0	0	0	0	0	0	0	14	165	56	0	0	0	0	0	0	0	0	0	0
S	240	0	0	0	0	0	0	44	0	0	37	160	0	0	18	7	0	0	0	5	0	0
L	241	0	151	20	22	0	4	0	44	0	0	0	4	0	0	16	0	0	0	0	10	0
S	242	0	0	0	0	44	0	0	0	0	24	165	10	0	3	13	0	0	4	0	10	0
P	243	0	0	0	0	37	0	0	7	44	17	0	0	0	0	10	0	142	0	20	0	0
A	244	34	7	0	8	10	0	0	44	0	14	0	4	0	114	4	12	0	6	13	0	0
W	245	10	20	48	0	0	0	10	30	0	0	0	0	0	0	0	152	0	0	0	4	0
F	246	0	4	32	140	0	14	30	0	0	0	3	4	0	0	0	0	0	0	54	0	0
E	247	19	2	0	0	0	0	0	0	0	7	0	0	0	10	12	44	18	22	145	2	0
A	248	44	135	59	10	0	0	0	0	10	0	12	5	0	5	0	0	0	0	0	1	0
L	249	20	0	30	72	0	11	0	0	0	3	0	0	0	0	0	0	145	0	0	0	0
K	250	3	0	16	14	0	10	10	0	15	0	0	29	0	0	5	0	144	20	0	0	0
M	251	11	0	0	0	0	31	0	0	0	144	0	0	3	0	0	22	20	4	0	0	4
V	252	8	27	2	168	0	0	0	0	9	0	0	0	3	0	0	0	6	0	0	0	0
L	253	0	0	52	10	0	14	0	0	0	0	0	0	140	0	7	0	0	0	0	0	0
E	254	3	13	9	0	0	0	0	0	0	0	2	0	1	0	140	16	23	7	0	1	0
K	255	12	0	0	2	0	0	0	0	0	0	4	128	7	10	0	2	1	33	3	0	0
I	256	0	0	17	14	0	0	16	0	1	0	0	85	7	0	0	0	0	0	1	0	0
G	257	0	0	0	0	0	0	0	0	20	23	0	0	7	0	0	1	0	0	0	0	0
F	258	0	0	0	0	0	0	18	0	0	0	0	0	3	0	0	0	0	0	0	0	0
G	259	0	0	0	0	0	0	0	0	14	0	0	0	0	0	0	0	0	0	0	0	0
D	260	2	0	0	0	0	0	0	0	0	0	0	0	0	2	0	14	0	0	0	0	0
R	261	0	0	0	0	0	2	0	0	2	0	0	0	0	0	0	0	0	0	14	0	0
V	262	0	46	0	2	5	0	0	0	0	7	3	0	0	0	13	0	0	4	2	0	0
D	263	2	13	0	0	0	0	0	10	0	2	5	3	0	7	12	14	0	32	0	0	0
Y	264	4	13	44	12	0	0	3	0	0	0	10	0	17	0	0	0	0	0	0	2	0
I	265	13	0	12	37	2	0	32	0	0	0	7	0	0	0	10	0	0	0	0	0	0
D	266	2	3	0	10	0	0	0	0	0	2	0	0	0	8	2	26	50	10	0	0	0
Y	267	3	0	4	0	0	0	0	0	0	2	42	0	17	10	0	16	0	0	10	7	0
L	268	0	2	63	2	0	5	0	0	0	31	0	0	2	0	0	0	0	0	0	3	2
N	269	0	13	38	3	0	10	0	0	0	3	6	15	0	13	2	0	0	0	0	0	0

The first column contains the amino acid of the query sequence at the position indicated in the second column. Each row in the table comprises a tally of the amino acids of each type (including non-standard indicated by 'X') from the corresponding positions in all qualified template fragments (positional hits). The following heading from the output provides the total number of templates searched plus other run parameters. Number of all PDB chains (SEQRES.all_list.11_03_09): 147,634; Structure: Polio RNApolym; Length: 461; MIN span: 5; Structure similarity: 55; LGA distance: 4.0; Window: 90; Legend: 's' AA reference sequence, 'd' dominant amino acid, '+' number of hits per position (max '+': n = 398).

**Table 2:** Excerpt of sample StralSV output "profile" file for positions 229-269 from poliovirus RdRp analysis

#	AA	ord	Rname	rnk	s/+	d/+	2nd/+	3rd/+	+/n	+	nv	variety
LSV_P:	L	229	229	2	14.7	72	14.7	7	35.9	143	6	GLIAHF
LSV_P:	F	230	230	1	81.9	81.9	5.2	3.2	38.9	155	7	FMYVLXI
LSV_P:	A	231	231	2	19.1	73.9	19.1	3.2	39.4	157	5	SAYDC
LSV_P:	F	232	232	3	13.2	54.5	19.1	13.2	64.6	257	8	YTFVALDI
LSV_P:	D	233	233	1	100	100	0	0	68.3	272	1	D
LSV_P:	Y	234	234	2	21.7	55.1	21.7	17.8	69.3	276	5	TYVIL
LSV_P:	T	235	235	3	9.1	52.2	33	9.1	69.3	276	6	RSTKAE
LSV_P:	G	236	236	4	8.3	50.4	17.4	8.3	69.3	276	9	CDAGNRKQX
LSV_P:	Y	237	237	4	10.1	55.1	15.9	13.4	69.3	276	7	FHWYCAG
LSV_P:	D	238	238	1	97.1	97.1	1.4	1.4	69.3	276	3	DYE
LSV_P:	A	239	239	3	14.9	59.8	20.3	14.9	69.3	276	4	STAG
LSV_P:	S	240	240	3	13.7	59	16.2	13.7	68.1	271	6	TFSNQR
LSV_P:	L	241	241	4	7.4	55.7	16.2	8.1	68.1	271	8	VWILQHMC
LSV_P:	S	242	242	3	8.8	60.4	16.1	8.8	68.6	273	8	TPSQCHKN
LSV_P:	P	243	243	3	13.4	51.3	15.9	13.4	69.6	277	7	EGPRSQW
LSV_P:	A	244	244	3	12.6	42.2	16.3	12.6	67.8	270	12	NWASRDPIVKCQ
LSV_P:	W	245	245	3	10.9	55.5	17.5	10.9	68.8	274	7	DLWVAFH
LSV_P:	F	246	246	4	10.7	49.8	19.2	11.4	70.6	281	8	IRLFMVCT
LSV_P:	E	247	247	5	6.4	51.6	15.7	7.8	70.6	281	10	RDKAEQNSVH
LSV_P:	A	248	248	3	15.7	48	21	15.7	70.6	281	9	VLATIGCNH
LSV_P:	L	249	249	3	10.7	51.6	25.6	10.7	70.6	281	6	EILAMS
LSV_P:	K	250	250	3	7.5	54.1	10.9	7.5	66.8	266	10	ECKLGIMFQA
LSV_P:	M	251	251	2	13	60.3	13	9.2	60.1	239	8	SMDEAKXY
LSV_P:	V	252	252	2	12.1	75.3	12.1	4	56	223	7	IVGAEYL
LSV_P:	L	253	253	2	23.3	62.8	23.3	6.3	56	223	5	YLMIQ
LSV_P:	E	254	254	2	10.7	65.1	10.7	7.4	54	215	10	QEDVLKATYH
LSV_P:	K	255	255	2	16.3	63.4	16.3	5.9	50.8	202	10	CKANYTRIDE
LSV_P:	I	256	256	4	9.9	60.3	12.1	11.3	35.4	141	7	CLFIYGR
LSV_P:	G	257	257	2	39.2	45.1	39.2	13.7	12.8	51	4	SGYD
LSV_P:	F	258	258	1	85.7	85.7	14.3	0	5.3	21	2	FY
LSV_P:	G	259	259	1	100	100	0	0	3.5	14	1	G
LSV_P:	D	260	260	1	77.8	77.8	11.1	11.1	4.5	18	3	DAN
LSV_P:	R	261	261	1	77.8	77.8	11.1	11.1	4.5	18	3	RMG
LSV_P:	V	262	262	1	56.1	56.1	15.9	8.5	20.6	82	8	VQSPKTIR
LSV_P:	D	263	263	2	14	32	14	13	25.1	100	10	KDVQWNTCAS
LSV_P:	Y	264	264	2	16.2	41.9	16.2	12.4	26.4	105	8	LYVITAFH
LSV_P:	I	265	265	1	32.7	32.7	28.3	11.5	28.4	113	7	IFALQTP
LSV_P:	D	266	266	2	23	44.2	23	8.8	28.4	113	9	EDIKNVASQ
LSV_P:	Y	267	267	2	15.3	37.8	15.3	14.4	27.9	111	9	TYDNRHLAS
LSV_P:	L	268	268	1	57.3	57.3	28.2	4.5	27.6	110	8	LSMHVIYX
LSV_P:	N	269	269	4	12.6	36.9	14.6	12.6	25.9	103	9	LCVNMTISQ

#: file name; AA: query amino acid; ord: sequence position number; Rname: PDB residue name; rnk: rank (position of the query amino acid in the "variety" list); s/+: percent of all residues corresponding to query; d/+: percent of dominant residue; 2^nd^/+: percent of second most dominant residue; 3^rd^/+: percent of third most dominant residue; +/n: percent of total templates selected in given experiment contributing to data at a given position; +: total number of templates contributing to data at a given position; nv: total number of amino acid variants; variety: list of variants in order of frequency.

### Selection of parameters for StralSV using benchmark structure set

**Figure 2:**
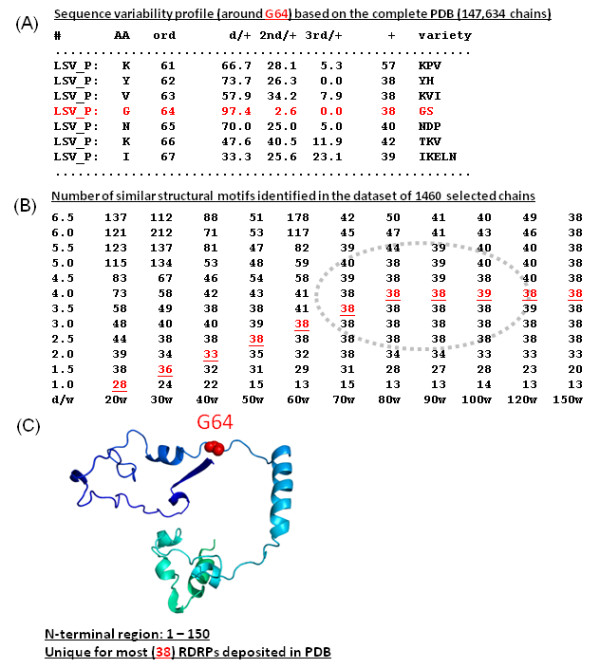
**Effect of window size and distance cutoff parameter combinations on capture of structure fragments**. A: sequence variability profile around position G64 generated by running StralSV against the PDB database; red type: data for position G64. B: Numbers of structure fragments selected from a custom library comprising 38 polymerase chains plus randomly selected structures from PDB; red type: numbers corresponding to distance cutoff and window_size values selected as defaults for StralSV web service. C: Structure fragment of poliovirus polymerase (1ra6) corresponding to the N-terminal region containing position G64; color variations dark blue to bright turquoise indicate N-to-C-terminal direction of chain.

To illustrate the StralSV algorithm, we selected a minimum span length of 5 and conducted analyses on poliovirus RdRp because poliovirus has been extensively studied and its polymerase is a member of a widely distributed protein family. In analyzing poliovirus RdRp, part of our effort involved determining suitable parameters for window_size and distance_cutoff. As window_size is increased, the stringency with which similar structure fragments are selected is increased, due to the greater structural context that is provided by the query segment. As distance cutoff is increased, that stringency is reduced, because more laxity in the alignment is allowed. Thus, for larger window_size values, a larger distance cutoff should be applied in order for the algorithm to not eliminate related structure fragments that have local structural deviations with respect to the query segment. Likewise, as the window size is decreased, the distance cutoff should be reduced as well, to prevent capture of many small, less related structure fragments. To determine what combinations of window_size and distance_cutoff values would provide comparable results, we performed several benchmark tests involving various structure libraries (e.g., complete PDB, PDB-select 40, ASTRAL40) and input parameters (data not shown). The results from one such test involving the capture of structure fragments from a benchmark data set containing 38 polymerase structures plus more than 1000 other structures randomly selected from the PDB is presented in Figure 2. Suitable parameters input to StralSV were expected to capture only structurally related fragments (i.e., the polymerases), whereas overly stringent parameters were expected to yield result sets lacking at least some of the polymerase fragments, and low stringency parameters were expected to capture less related fragments. In this way, we examined the dependency between window_size and distance_cutoff values to determine optimal (for the current study) parameter settings, and help define default parameter settings for StralSV.

We conducted a test whereby window_size and distance_cutoff parameters were varied from 20 to 150 and 1.0Å to 6.5Å, respectively (Figure 2). We ran StralSV using each window_size/distance_cutoff combination for all query (poliovirus RdRp) segments enclosing residue G64. We selected a region in the N-terminal portion of the protein upon which to focus this exercise. The region defined by residues 1-140 tended to be structurally unique, but at the same time contained a variety of well defined secondary structure elements of different sizes (Figure 2C). Furthermore, the selection of fragments to inspect from within this region was somewhat arbitrary; we selected those segments containing residue G64 due to its significance as a functional residue [18]. We determined how many qualified hits were obtained for each window_size/distance_cutoff combination (Figure 2B). Parameter settings comprising window_size values ranging from 70 to 90 at distance cutoffs 3.0Å to 5.0Å gave satisfactory results; fragments from the seeded 38 polymerase structures were reliably captured within a set of window_size and distance_cutoff parameter value combinations within these ranges (dotted oval in Figure 2B). Window_size values smaller than 70 tended to yield qualified hit sets missing some of the 38 polymerases (i.e., true positives) for lesser (more stringent) values of distance_cutoff and tended to capture increasing numbers of unrelated (false positive) structure fragments as the distance cutoff was increased. Also, window_size values smaller than 70 were sensitive to the distance cutoff value, yielding acceptable result sets only for narrow ranges of distance cutoff. Very large window_size values (e.g., 150) resulted in selection of all true positives for all but the very tightest (distance_cutoff < 2.5Å) alignments. The parameter settings highlighted in red type in Figure 2B were selected as default values for StralSV.

### Analysis of poliovirus RdRp

#### Parameters

Based on parameters applied in Zemla et al. 2007 [17] and the analysis described above, we used StralSV to analyze sequence variability in structure context for poliovirus RdRp (PDB: 1ra6; [19]) using minimum span length of 5, LGA_S cutoff 55%, and window_size/distance_cutoff combinations 50/2.5Å, 70/3.5Å, 80/4.0Å, and 90/4.0Å.

#### Plotting of StralSV results

**Figure 3:**
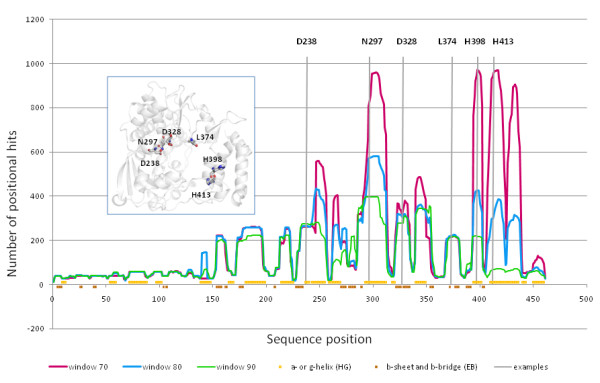
**Effect of window size on numbers of positional hits per position**. Red: window size 70; blue: 80; green: 90. Vertical lines with labels: positions at which SCOP identifiers were quantified for templates contributing to positional variability data (see Table 3 and additional files 3, 4, 5, 6, 7, and 8: StralSV-RdRp_Suppl_Table2, StralSV-RdRp_Suppl_Table3, StralSV-RdRp_Suppl_Table4, StralSV-RdRp_Suppl_Table5, StralSV-RdRp_Suppl_Table6, StralSV-RdRp_Suppl_Table7). Along x-axis: secondary structure assignments (see Methods). Inset: structure model of poliovirus polymerase upon which have been labeled the six positions highlighted in the main figure.

**Figure 4:**
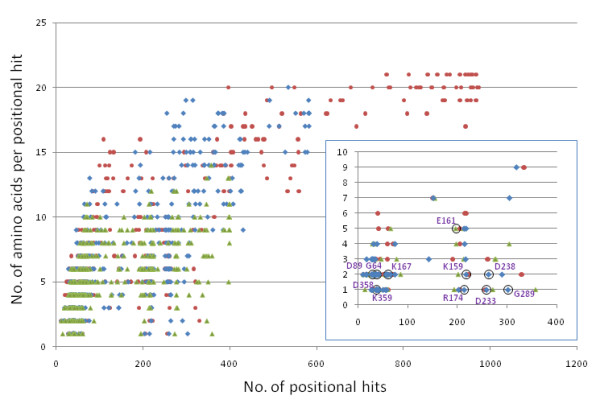
**Amino-acid variability (y-axis) versus number of positional hits (x-axis) for three window sizes**. Red circles: window size 70; blue diamonds: 80; green triangles: 90. Inset: positional hits at which the dominant residue occurs at frequency > = 80%. Circles: positional hit, variability coordinate pairs corresponding to the 11 positions shown in Fig. 5.

**Figure 5:**
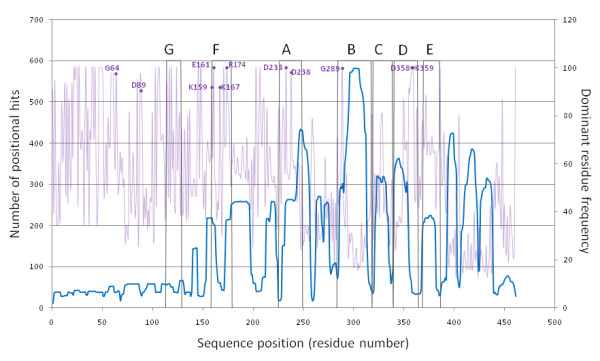
**Frequencies of dominant residues and correspondence of positional hit frequency with polymerase sequence motifs A-G**. Lavender plot: frequencies (quantified along right y-axis) of dominant residue per sequence position along poliovirus polymerase chain; blue plot: positional hit frequency at window size 80; labeled dots: high-frequency residues that have been functionally annotated (see additional file 2: StralSV-RdRp_Suppl_Table1).

StralSV produced variability matrices and sequence profiles (not shown; for excerpts see Tables 1 and 2) from which were extracted data for analyzing related structure fragments and quantifying positional variability. Plots containing data extracted directly from the matrix and profiles files are shown in Figures 3, 4, and 5. We performed additional analyses in order to annotate the primary StralSV results: 1) Secondary structure assignments were calculated for poliovirus RdRp using DSSP [20] and were plotted along the x-axis of Figure 3. DSSP output was simplified as follows: helix, comprising alpha helix (H), pi helix (I), and 3/10 helix (G); strand, comprising extended strand (E) or residue in isolated beta bridge (B). 2) To determine whether we could discern patterns with respect to positional hit frequency and previously identified polymerase sequence motifs, we overlaid a plot comprising qualified hits versus sequence position with the positions of the well known motifs, extracted from the literature (Figure 5, gray boxes with labels A-G). This was accomplished by examining sequence alignments and extracting coordinates defining the known sequence motifs A through G from papers that included poliovirus RdRp among the aligned sequences [21-24]. Because there was considerable inconsistency regarding the boundaries of the sequence motifs, we defined inclusive boundaries for each motif whereby residues were included if they were identified within a motif in any of the sequence alignments reported in the literature. 3) In order to inspect the most abundant sequence variants (i.e., the most conserved positions) in the context of the positional hit frequency along the reference protein, we extracted from the matrix files the frequencies of the most frequent residues and plotted them versus sequence position along with the positional hits for window size 80 (Figure 5). 4) The literature was searched for evidence of functional annotation [18,21-31] for the most frequently observed residue positions (see additional file 2: StralSV-RdRp_Suppl_Table 1) and positions for which functional annotation was identified were marked in Figure 5.

#### Annotation of representative positional hits

The qualified hits (structure fragments from PDB with detected local similarities to the query structure) for six selected sequence positions (positional hits) of polio RdRp (positions identified in Figure 3) were categorized and quantified based on SCOP (Structure Classification of Proteins database; version 1.75, June 2009 release) identifiers [32]. Note that because SCOP is a manually curated database of structure domains from the PDB, there is some delay (currently more than one year) before a new PDB entry is classified in SCOP. Therefore, we have included in Table 3 and additional files 3, 4, 5, 6, 7, and 8 (StralSV-RdRp_Suppl_Table 2, StralSV-RdRp_Suppl_Table 3, StralSV-RdRp_Suppl_Table 4, StralSV-RdRp_Suppl_Table 5, StralSV-RdRp_Suppl_Table 6, StralSV-RdRp_Suppl_Table 7) data pertaining to qualified hits and sequence variability based on StralSV analysis using a complete tally of PDB identifiers in addition to those hits and variabilities that could be categorized by SCOP classification. The hits were evaluated separately for each of windows 50, 70, 80 and 90, at six sequence positions selected to be representative of the types of frequency variation (of qualified hits) over the length of the polymerase (see specified positions in Figure 3). For each of the six selected positions, the following information was extracted from the StralSV matrix output file: all PDB templates (including chain information) that contributed to the profile at that position, the corresponding LGA_S score, the sequence identity (Seq ID), the number of (template) amino acids that matched the query, the name of the corresponding amino acid of the template, and, when available, the SCOP identifier for each classified PDB template.

**Table 3:** SCOP categorization of template fragments selected at each of six positions along poliovirus RdRp

		RdRp		DsPhage	RT	DNA pol1	Other	Templates
Position	Window	e.8.1.4		e.8.1.6	e.8.1.2	e.8.1.1	not e.8	with SCOP ID
D238	w90	85		32	0	0	3	120
	w80	85		32	0	0	0	119
	w70	79		32	0	0	0	111
	w50	79		0	0	0	0	79
N297	w90	85		32	78	0	9	204
	w80	85		32	41	2	160	320
	w70	85		32	71	3	289	480
	w50	78		32	0	4	197	311
D328	w90	84		32	3	0	6	125
	w80	84		32	8	0	17	141
	w70	84		32	4	0	34	154
	w50	71		2	0	0	3	76
L374	w90	77		0	0	0	2	79
	w80	80		0	0	0	3	83
	w70	82		0	1	0	3	86
	w50	59		0	0	0	2	61
H398	w90	79		0	0	0	3	82
	w80	80		0	0	0	48	128
	w70	68		0	0	**2**	376	446
	w50	36		0	0	0	182	218
H413	w90	37		0	0	0	1	38
	w80	45		0	0	0	54	99
	w70	40		0	0	2	392	434
	w50	37		0	0	13	695	745

Position: residue and position number in poliovirus polymerase; Window: window_size parameter settings (w: window); RdRp e.8.1.4: RNA-dependent RNA polymerase; DsPhage e.8.1.6: dsRNA phage RNA-dependent RNA-polymerase; RT e.8.1.2: Reverse transcriptase; DNA pol1 e.8.1.1: DNA polymerase I; Other not e.8: all other SCOP families.

KEY:

e.8.1.4 RdRp RNA-dependent RNA-polymerase

e.8.1.6 DsPhage dsRNA phage RNA-dependent RNA-polymerase

e.8.1.2 RT Reverse transcriptase

e.8.1.1 DNApol1 DNA polymerase I

## Results

### Effects of parameter settings on capture of database structure fragments

Numbers of positional hits (residues within spans) corresponding to each position along the poliovirus query structure were plotted for window_size/distance_cutoff parameter pairs 70/3.5Å, 80/4.0Å, and 90/4.0Å (hereafter referring only to the window_size) (Figure 3). In some regions, there was observed considerable variation in the numbers of positional hits captured for the different window_size settings (e.g., positions within ranges 290-310 and 390-440), whereas at other positions, window_size had little effect on the numbers of qualified hits captured (most notably positions within ranges 1-240 and 360-390). Observations of positional hit frequency variations over the length of poliovirus RdRp led to identification of a variability profile (illustrated in Figures 3 and 5). The regions of high variability were "locked" between regions of high specificity, which exhibited little or no change with the variation of window_size. As window_size was increased, the numbers of positional hits tended to decrease in those regions for which window_size-dependent variability was observed. Comparison of positional hit variabilities to secondary structure (Figure 3, along x-axis) revealed that the window-related variability tended to occur in regions of helical secondary structure. A polymerase "baseline", amounting to fewer than 50 positional hits (corresponding primarily to polymerase structures) was observed along the entirety of the poliovirus chain (Figure 3 x-axis). All polymerase baseline regions, with the exception of region 410-461, were identifiable independently of window_size. The latter C-terminal region showed high specificity at window size 90. In this region, lowering the window_size value to just 70 resulted in the capture of many (> 900) structure fragments that have apparent structural similarity to poliovirus RdRp (Figure 3, tall red peaks at right side of plot). The N-terminal region of the poliovirus protein yielded the least positional hits, indicating relative structural uniqueness in this region.

### Composition of captured structure fragments at specific positions

We selected six positions (Figure 3, residues numbered at top of plot) at which to examine the diversity of structure fragments captured at each of four window_size values (Table 3, additional files 3, 4, 5, 6, 7, and 8: StralSV-RdRp_Suppl_Table 2, StralSV-RdRp_Suppl_Table 3, StralSV-RdRp_Suppl_Table 4, StralSV-RdRp_Suppl_Table 5, StralSV-RdRp_Suppl_Table 6, StralSV-RdRp_Suppl_Table 7). Orientations within the structure of poliovirus RdRp of these six positions are indicated on the structure model shown in the inset of Figure 3. Qualified hits were examined for positions at which numbers of positional hits were relatively invariant (D238, D328, L374) or highly variant (N297, H398, H413) among the calculations run at the four window_size values. (In some cases preferences for selection of a particular residue for this analysis was based on existence of a functional annotation for that residue and, therefore, of biological interest (e.g., D238, N297; [21,29]). We included window_size 50 in this analysis in order to determine what effect a very small (low stringency) window_size value might have on the structure-function diversity of qualified hits (i.e., would it greatly increase the diversity?). For each position and each window_size, qualified hits were categorized by SCOP concise classification strings. Hits were categorized into four families in the e.8.1 (DNA/RNA polymerases) superfamily: e.8.1.1 (DNA polymerase I), e.8.1.2 (Reverse transcriptase), e.8.1.4 (RNA-dependent RNA-polymerase), and e.8.1.6 (dsRNA phage RNA-dependent RNA-polymerase), and into various other non-e.8 SCOP classifications.

At positions D238, D328, and L374, qualification of structure fragments (and resulting positional hits) was largely independent of window_size, and few unrelated (non-e.8) fragments were captured, indicating that the structure motifs in the surrounding regions were largely limited to the families detected (e.g., e.8.1.4 and e.8.1.6) (Table 3, positions D238 and D328). For positions N297, H398, and H413, at which there was considerable diversity in the SCOP families detected at smaller window_size values (< 90), omission of these more distantly related positional hits was observed as window_size increased. Overall it was observed that window_size 90 resulted predominantly in capture of structure fragments from the structure family to which poliovirus RdRp belongs (e.8.1.4). Therefore, at all six positions, window_size 90 effectively filtered from among the nearly 150,000 PDB chains only those members of the polymerase/transcriptase families.

### Detection of sequence variability in structure context

To determine how sequence variability was distributed among the e.8.1 families, we further categorized the amino-acid variabilities by SCOP family for position N297 at window_size 80 (Table 4). In general approximately 2/3 of the positional hits could be categorized using SCOP; as an example, 320 of the 572 total positional hits were derived from structures that had been classified in SCOP. For this position (at which the greatest sequence variability was observed among the 6 selected), the distribution of amino-acid variability contributed by e.8 superfamily members (e.8.1.1, 2, 4, and 6) was considerably more narrow (5 amino acids (A, F, G, H, N), of which half (80) were N) than that observed overall (13 amino acids). All of the positional hits that coincided in sequence with poliovirus RdRp at N297 were members of the e.8.1.4 (RNA-dependent RNA-polymerase) family, although a minority (5; all of the remaining) of the hits (all RdRp of lambda 3) from this family had H at position 297. Thus, sequence variability was limited to N (94%) and H (6%) within family e.8.1.4, in which poliovirus RdRp is categorized. (This family also includes HCV, FM, lambda 3, BVDV, Norwalk virus, rhinovirus, rabbit hemorrhagic fever virus, and IBVD.) Summarizing the amino-acid occurrences among qualified hits within fold e.8 (comprising 1 superfamily and 4 families), we observed the following amino-acid distributions at position N297: N = 50%, F = 26%, G = 20%, H = 3%, A = 0.6%, compared to a much broader distribution of amino-acid variabilities observed within templates outside of fold e.8: V = 31%, R = 31%, Q = 9%, I = 6%, H = 5%, Y = 5%, and C, E, F, K, L, M, and T each < = 3% (comprising the "tail" in a distribution of sequence variability). For completeness, amino-acid variabilities derived from positional hits at all six positions examined (see Figure 3) are summarized in additional files 3, 4, 5, 6, 7, and 8: StralSV-RdRp_Suppl_Table 2, StralSV-RdRp_Suppl_Table 3, StralSV-RdRp_Suppl_Table 4, StralSV-RdRp_Suppl_Table 5, StralSV-RdRp_Suppl_Table 6, StralSV-RdRp_Suppl_Table 7. Distributions of observed amino-acids are shown for all templates and grouped as within or outside of fold e.8. From this detailed analysis it was possible to categorize the specific amino-acid variabilities per position--thus, StralSV can be used to detect positional trends and anomalies among structures related to the protein of interest.

**Table 4:** Effect of window_size on sequence variability in SCOP-categorized structure fragments at position N297.

Position: 297, Window: 80, Categories: 33, PDB templates: 399, SCOP_IDs: 320							
**Templates**	**all PDB templates**	**e.8 templates**	**RNA-dependent RNA polymerase**	**dsRNA phage RNA-dependent RNA polymerase**	**DNA polymerase I**	**Reverse transcriptase**	**Templates not e.8**

ScopID		e.8	e.8.1.4	e.8.1.6	e.8.1.1	e.8.1.2	

max LGA		100.0	100.0	76.9	57.0	59.2	76.8

ave LGA		75.4	85.2	75.1	57.0	56.4	57.8

ave ID		27.3	37.3	13.4	3.7	18.6	6.2

matches		51	61	40	31	39	35

AA	572	160	85	32	2	41	160

A	1	1			1		

C	8						3

D	1						

E	3						1

F	67	42			1	41 ^1^	5

G	44	32		32 ^2^			

H	14	5	5 ^3^				8

I	19						9

K	2						2

L	11						5

M	2						2

N	224	80	80 ^4^				

P							

Q	15						14

R	82						49

S							

T	15						4

V	54						50

W							

Y	10						8

X							

^1^F297 (N = 41): reverse transcriptase of HIV

^2^G297 (N = 32): dsRNA phage RNA-dependent RNA polymerase of phi6

^3^H297 (N = 5): RdRp of lambda 3

^4^N297 (N = 80): RdRp proteins from HCV (21), FM (8), lambda3 (5), polio (6), BVDV (3), Norwalk virus (3), rhinovirus (3), rabbit hemorrhagic fever virus (2), IBDV (1)

### Effect of window_size on detection of sequence variability

To determine how the window size parameter might affect the detection of amino-acid variability "globally" along the poliovirus RdRp chain, we plotted for each position along the reference structure the detected absolute amino-acid variability versus the number of qualified hits for window_size values 70, 80, and 90 (Figure 4). As window_size decreased from 90 to 70, there was observed an increase in the number of database structure fragments that contributed positional hits, and a corresponding increase in the amino-acid variabilities: at window_size 70 there were far more (32) positions at which all amino-acids were observed among the positional hits compared to window_size 80 (1 position). At window_size 90 there was considerably less sequence variability detected among the positional hits, with the most variable positions accounting for no more than 14 distinct amino acids observed. The inset in Figure 4 displays the data points for those positions at which the dominant (most frequently observed) amino-acid occurred at a frequency of at least 80%. Circled data points are those corresponding to window_size 80. There was considerable overlap among window_size values 70, 80, and 90 with respect to positions at which the dominant residue occurred at frequency > = 80%. Window_size 70 produced 79 positions, 80 produced 74, and 90 produced 79; 62 positions occurred in all three of these data sets. This plot demonstrates that the most dominant amino-acid residues occurred within the same (narrow) positional hit frequency range (0 to < 400) regardless of window_size, suggesting that highly conserved positions display relatively little sequence variability regardless of the selected window_size.

### Sequence and structure motifs associated with RdRps

Regions corresponding to the well-known sequence motifs characteristic of polymerases were mapped to the positional hit frequency plot for window size 80 (Figure 5). As mentioned above, regions of high positional hit frequency tended to correspond with helix secondary structure (Figure 3), but also with defined palm-domain motifs (Figure 5). Also plotted in Figure 5 are the frequencies of the dominant residues per position along poliovirus RdRp (lavender plot in figure). Included are residue-position labels for those positions at which the dominant residue frequency exceeded 90% and for which we were able to identify a functional annotation in the literature (see additional file 2: StralSV-RdRp_Suppl_Table 1). Although there is no clear criterion for identifying functional residues and short sequence or structure motifs based on StralSV profiles, it was evident that functionally relevant residues tended to emerge when selecting those positions displaying high degrees of conservation in structure context. Furthermore, lowering the window size to just 70 resulted in the capture of many structure fragments with common structure motifs (see last 3 maxima in Figure 3 graph and Table 3 "other" column for positions H398 and H413), implying that StralSV may enable identification of common structure motifs shared among distantly related proteins.

## Discussion

StralSV differs in several respects from other sequence- and structure-based algorithms for comparing proteins. First, by applying an overlapping sliding window to define segments of a structure of interest, the algorithm avoids the pitfalls of algorithms that compare proteins at a global level: by dividing a protein into segments, StralSV enables comparison of structures by using fragments corresponding approximately in size to super-secondary elements or structure motifs. In this way, portions of a structure can be compared at a local level to like fragments in the PDB without losing the greater structural context. StralSV applies a two-step approach to filtering PDB fragments in order to select those which are most likely to be relevant for a meaningful comparison. For example, the results presented in Tables 3 and 4 and additional files 3, 4, 5, 6, 7, and 8: StralSV-RdRp_Suppl_Table 2, StralSV-RdRp_Suppl_Table 3, StralSV-RdRp_Suppl_Table 4, StralSV-RdRp_Suppl_Table 5, StralSV-RdRp_Suppl_Table 6, StralSV-RdRp_Suppl_Table 7, illustrate how StralSV can be used to quantify and annotate sequence variability among structure fragments that form tight local alignments (determined by span and distance_cutoff parameters), yet have sufficient structure context (determined by window_size) to filter out unrelated fragments. Larger window_size values effectively increase the stringency with which structure fragments are selected, whereas smaller distance_cutoff values also increase this stringency, but more locally, by enforcing tighter local alignments. As seen in Table 3, considerably fewer fragments are selected at window_size 90 than with smaller window_size values for those positions that are sensitive to window_size, but the result set is greatly enriched for fragments that are closely related to the reference structure (i.e., polio RdRp). How much stringency the user wishes to apply in running StralSV may depend on one's research interest and the protein being studied. Smaller window_size (as well as greater distance_cutoff) parameter values will result in capture of more structure fragments, many of which will be from structures that are more distantly related in terms of their SCOP classification and the taxonomy of the organisms that they are from (Table 5). It must be noted that as the window_size (or distance_cutoff) parameter is relaxed, more "noise" arises in the result set; however, at the same time there is potential for discovering structural relationships and sequence laxity that may not otherwise be discernable when comparing only closely related structures.

**Table 5:** Taxonomic diversity represented by positional hits detected at position N297.

window	50	70	80	90
No. of PDBs	189	198	199	141
ARCHAEA	+	+	+	
BACTERIA	+	+	+	+
BIRD	+			
FUNGUS	+			
INSECT	+	+	+	
MAMMAL	+	+	+	+
PHAGE	+		+	+
VIRUS	+	+	+	+
WORM	+	+		
YEAST	+	+	+	

### Effects of parameter setting on capture of database structure fragments

Not unexpectedly, the frequencies of positional hits tend toward maxima in regions of helical secondary structure, and as window_size is decreased the numbers of positional hits tend to increase (Figures 3, 5). Reduction in window_size relaxes constraints on the alignment, and therefore smaller fragments may align more tightly, thereby meeting the distance cutoff. In some regions, the numbers of positional hits do not change significantly with changes in window_size (e.g., peaks up to position 240 and 360-390), indicating that in certain regions there exist conserved structure motifs that are shared only among a specific set of structures. For example, there is observed in the N-terminal regions up to approximately position 140 (Figures 3, 5) a "polymerase baseline", which appears to be structurally unique. This region is external to the catalytic tunnel of the polymerase, and may define structural and functional specificity for polio and related viruses. Window_size 90 produced a polymerase baseline in the C-terminal region as well, although here the positional hit frequency was highly sensitive to window_size. It may be that only window_size 90 (or greater) effectively filtered out non-polymerase structure fragments due to the stringency enforced by context, as it appeared that smaller segments of polio RdRp in this region resembled short segments in distantly related proteins.

We observed considerable diversity among the SCOP families represented by fragments detected at different positions at window_size values less than 90 (N297, H398, H413). This demonstrated a clear filtering of these positional hits as the window_size increased. One must be aware, however, that the two parameters, window_size and distance_cutoff, have an opposing relationship with respect to qualifying a hit: increase in alignment stringency is achieved by increasing window_size, but also by decreasing distance_cutoff. As window_size is decreased, it is necessary to also reduce the distance_cutoff in order to avoid an unacceptable increase in the number of "false positive" (considerably less structurally relevant) fragments captured. We achieved a reasonable selection of window-size/distance-cutoff parameter pairings by examining the relationship between these parameters (Figure 2). The apparent (perhaps unexpected) decrease in the numbers of non-e.8 structure fragments captured by window 50 compared to window 70 at positions N297 and H398 (Table 3 "Other" column) are explained by the high-stringency filtering achieved by the distance cutoff 2.5 Å applied at window 50. Thus, it is desirable to strike a balance between window size and distance cutoff. Small window_size values are useful for capturing shorter fragments, but to ensure that the result set is not populated by spurious hits corresponding to ubiquitous secondary structure elements (e.g., alpha helix), one must apply added stringency at the level of distance_cutoff. Smaller window_size values are appropriate when the user is interested in focusing the analysis on a relatively small structure motif, which may occur with or without the surrounding structural context in the reference structure. In capturing these smaller structure fragments, one is cautioned to enforce tighter alignments in order to assure that the resulting qualified hits are relevant to the study.

### Detection of Sequence Motifs

Regions corresponding to the well-known sequence motifs A-G, characteristic of polymerases, were mapped to the positional hit frequency plot for window size 80 (Figure 5). All categories of polymerase (RdRp, RdDp/RT, DdRp, DdDp) are recognized to contain sequence motifs A-D. Identification of motifs E, F, and G, however, has been somewhat obscured by the greater diversity among sequences assigned to these structure motifs. O'Reilley and Kao [23] reported motif E as being exclusive to RdDps and RdRps, and motifs F [22,24] and G [24] were identified in poliovirus RdRp. Motif F was identified in phi6 [33], BVDV and HCV [34], reovirus, phi6, BVDV, HCV, rhinovirus, Norwalk virus, and HIV [35], and FMDV, RHDV, and HIV1 [36]. A detailed structure-based comparison of these polymerases using StralSV may clarify the assignment of motifs A-G among the polymerase classes.

### Detection of sequence variability in structure context

In examining the amino-acid variability versus qualified hit frequency (Figure 4 inset, circled data points) for 11 highly conserved positions (Figure 5 dots) that had been functionally annotated (see additional file 2: StralSV-RdRp_Suppl_Table 1), it appeared that the numbers of positional hits and the degree of amino-acid variability observed for high-frequency positions was largely independent of window_size. This implies (at least for the positions that were examined in this study) that the functionally relevant positions are consistently detected as high-frequency-residue positions regardless of window_size. Therefore, the StralSV algorithm is sufficiently sensitive to detect structurally or functionally conserved residues even when the parameters may not be perfectly tuned.

StralSV is especially useful for identifying highly conserved residues at positions that occur in regions in which there are large numbers of positional hits; dominant residue frequency cannot be considered significant in regions of structural conservation (e.g., the N-terminal region up to about position 140 in poliovirus RdRp), whereas identification of dominant residues occurring with high frequency at positions with large numbers of positional hits may help identify residues at positions that are structurally and/or functionally significant. For example, more than 250 positional hits contributed to residue counts at positions D233 and D328 (window_size 80, Figure 5), known to be critical for RdRp function. Aspartic acid occurred at close to 100% frequency at these positions. The structure fragments contributing to these data points comprised RdRps and dsRNA phage polymerases (SCOP family e.8.1.4). The combination of many positional hits and low amino acid variability may provide a means of identifying key functional residues.

Because the ability to detect possibly functional residues is of particular interest in protein functional annotation, we compared the results obtained using StralSV to those of another bioinformatics tool [37]. FireStar uses alignments to identify functional residues based on close atomic contacts in PDB structures and annotated residues in the Catalytic Site Atlas (CSA). We ran poliovirus RdRp (PDB: 1ra6) through the online FireStar server (data not shown), and found considerable overlap between the functional residue list generated by FireStar and the list of 90% dominance residues generated by StralSV (see additional file 2: StralSV-RdRp_Suppl_Table 1): K158, K167, R174, D233, D238, G289, and K359. StralSV identified the more broadly conserved residues within the functional set identified by FireStar. A main difference between the two approaches is that FireStar identifies functional residues involved in ligand binding--some being highly conserved and others displaying considerable sequence variability. In particular we note N297, which is determined by FireStar to be associated with binding of cytidine-s-triphosphate and uridine-s-monophosphate (sites 2 and 3). StralSV identified N297 as a residue that displays some degree of conservation, but also quantified the degree of conservation at this position across a wide range of structures (see additional file 4: StralSV-RdRp_Suppl_Table 3). Furthermore, StralSV is not limited to identification of residues that have been associated with a binding site, but can be used to infer structure and/or functional significance based on sequence conservation in structure context regardless of pre-existing annotation information.

### Additional applications of StralSV

Results from StralSV analysis can be used to characterize residue positions in a reference protein by detection of similar locations in other proteins (sometimes from quite distant organisms and different assigned structure classifications), in which corresponding residue positions within similar structural motifs are observed. Such analyses may potentiate rapid identification of invariant (as well as unusual or unexpected) residues, which in many cases are essential to a protein's function. It also may enlighten studies of newly discovered natural or engineered mutations that have not yet been observed in the sequence databases. Results from StralSV structure similarity searches performed against large sets of structurally related proteins can facilitate refinement of constructed homology models by suggesting corrections to the query-template alignments (using an approach similar to that of [2]) or by providing a list of possible conformational variants of corresponding structural fragments for loop-building procedures. Calculated residue-residue correspondences can be used to evaluate pure sequence alignment methods and also to derive structural environment-specific substitution matrices, which have been shown to be useful for detection of remote homologs [38]. When applied to experimentally solved structures, StralSV also could facilitate identification of structural motifs (local conformations) that have not yet been observed in PDB. Such findings could aid in the discovery of previously unidentified structural motifs or suggest refinement of constructed structural models in particular regions.

## Conclusions

StralSV is a new algorithm for detecting closely related structure fragments from a structure database (PDB or user-defined) and quantifying residue frequency from tight local structure alignments. Input parameters to StralSV (window_size, distance_cutoff, or span_size) can be varied in order to adjust the stringency with which structure fragments are selected or with which local alignments are made, thereby providing the user with flexibility in detecting similar structure fragments. High-stringency parameter settings will effectively filter out all but highly structurally similar fragments and will impose very tight local alignments, whereas low-stringency parameters will enable detection of more distantly related structures, which may be of interest, for example, when the user wishes to detect distant evolutionary relationships among proteins or to test the range of possible sequence variability that might be expected to be tolerated within a given structure motif. It should be emphasized, however, that StralSV safeguards against degradation of sequence variability data quality by enforcing structure context upon local alignments in a two-step process of identifying "qualified" hits.

It has long been recognized that peptides with very different sequences may have similar tertiary structures. In this work we applied StralSV in a study of the RNA-dependent RNA polymerase of poliovirus and demonstrated that the algorithm could be used to determine regions of the protein that were relatively unique (e.g., the N-terminal region) or that shared structural similarity (e.g., C-terminal motifs) with structures that were distantly related (non-e.8 SCOP classifications), and that by quantifying residue frequencies among many (hundreds or even thousands) of residue-residue pairs extracted from local alignments, one can infer potential structural or functional importance of specific residues that are determined to be highly conserved or that deviate from a consensus. We further demonstrated that considerable detailed structural and phylogenetic information can be derived from StralSV profiles.

StralSV is available as a web service at http://proteinmodel.org/AS2TS/STRALSV/.

## Authors' contributions

AZ designed and developed the StralSV algorithm, performed calculations for poliovirus RdRp, and contributed the background material on structure-based methods. CZ and DL wrote codes and developed methods for post-processing of StralSV results, and performed literature searches for interpretation of biological significance of various residue positions of poliovirus RdRp. CZ and AZ wrote the manuscript, with contributions from DL and TK. All authors participated in the discussions and shaped the ideas that led to the experimental design and results of this work. All authors read and approved the manuscript.

## Supplementary Material

Additional file 1 **StralSV-RdRp_Suppl_Figure1**. Illustration of a span. Click here for file

Additional file 2 **StralSV-RdRp_Suppl_Table1**. Dominant residues and functional annotations taken from the literature. Click here for file

Additional file 3 **StralSV-RdRp_Suppl_Table2**. Effect of window_size on selections of fragments and sequence variability for position D238. Click here for file

Additional file 4 **StralSV-RdRp_Suppl_Table3**. Effect of window_size on selections of fragments and sequence variability for position N297. Click here for file

Additional file 5 **StralSV-RdRp_Suppl_Table4**. Effect of window_size on selections of fragments and sequence variability for position D328. Click here for file

Additional file 6 **StralSV-RdRp_Suppl_Table5**. Effect of window_size on selections of fragments and sequence variability for position L374. Click here for file

Additional file 7 **StralSV-RdRp_Suppl_Table6**. Effect of window_size on selections of fragments and sequence variability for position H398. Click here for file

Additional file 8 **StralSV-RdRp_Suppl_Table7**. Effect of window_size on selections of fragments and sequence variability for position H413. Click here for file
