# Supplementary material for: StralSV: assessment of sequence variability within similar 3D structures and application to polio RNA-dependent RNA polymerase
Source: BMC Bioinformatics. 2011 Jun 2;12:226. doi: 10.1186/1471-2105-12-226 (PMC3121648; doi:10.1186/1471-2105-12-226)

Suppl. Fig. 1. Illustration of a span. Plot shows a backbone representation of two superimposed structures (Molecule1 = 1jx7_A and Molecule2 = 1l1s_A), and structure deviations calculated using LGA. A span is a contiguous set of residues from two structures comprising a tight local alignment (L-RMSD <=0.5 Å), and length of at least 3 amino-acids. Three spans are identified as regions: 85-92 (size=8), 99-106 (size=8), and 109-112 (size=4). Spans are colored in green, and the residue ranges reflect residue numberings from 1l1s_A. Only residue-residue correspondences that occur within identified spans that meet the specified criteria (local RMSD and minimum span_size) contribute to the amino-acid counts in the matrix and profile output files.


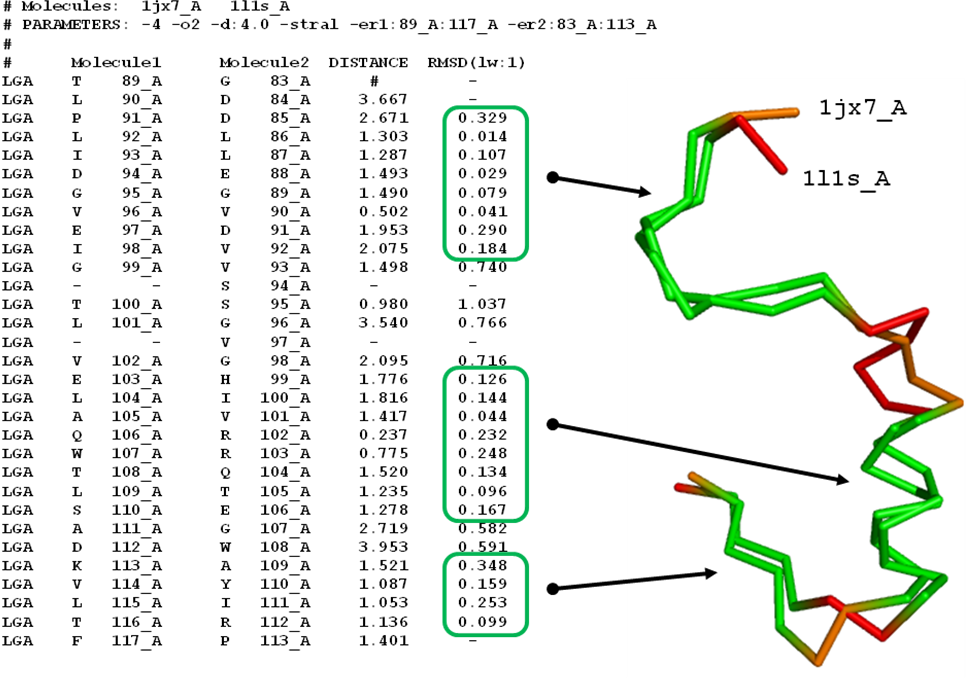

Supplement: Additional file 1 — StralSV-RdRp_Suppl_Figure1. Illustration of a span. [file 1471-2105-12-226-S1.DOC]
